# Development of TaqMan Real-Time Fluorescent Quantitative PCR Method for Identification and Quantification of *Sinomenium acutum-*Originated Herbal Drugs

**DOI:** 10.3390/molecules30183763

**Published:** 2025-09-16

**Authors:** Ye Tao, Shuchen Duan, Kunzi Yu, Xianlong Cheng, Xiangri Li, Wenjuan Zhang, Yazhong Zhang, Feng Wei

**Affiliations:** 1 School of Chinese Materia Medica, Beijing University of Chinese Medicine, Beijing 102488, China; 13856080281@163.com (Y.T.); lixiangri@sina.com (X.L.); 2 National Institutes for Food and Drug Control, Beijing 102629, China; dsc65432@163.com (S.D.); yukunzi@nifdc.org.cn (K.Y.); lncxl@sina.com (X.C.); 3 NMPA Key Laboratory for Quality Research and Evaluation of Traditional Chinese Medicine, Anhui Institutes for Food and Drug Control, Hefei 230051, China; 4 School of Chinese Materia Medica, China Pharmaceutical University, Nanjing 211198, China

**Keywords:** *Sinomenium acutum*, qPCR, adulterant, preparations, aqueous decoctions, quantification

## Abstract

Due to morphological similarities, adulterants are frequently substituted for Qingfengteng (QFT, *Sinomenium acutum*) in regional markets. This study developed a TaqMan probe-based real-time quantitative polymerase chain reaction (qPCR) assay targeting a 57-base pair (bp) fragment of the internal transcribed spacer 2 (ITS2) region for the specific detection of QFT. The method was validated using a diverse set of samples, including: (1) 19 batches of QFT and 8 batches of Beidougen (BDG, *Menispermum dauricum*), comprising both medicinal materials and decoction pieces; (2) 5 batches each of decoction pieces from Qingsheteng (QST, *Periploca calophylla*), Jishiteng (JST, *Paederia foetida*), Kuanjinteng (KJT, *Tinospora sinensis*), and Huibeiqingfengteng (HBQFT, *Sabia discolor*); (3) 6 batches of commercial QFT-containing tablets (with different batch numbers) and 6 batches of laboratory-prepared QFT aqueous decoctions (with different decocting time). Distinct cycle threshold (Ct) values and amplification curves unambiguously differentiated QFT from all adulterants. An external standard-based quantification approach was established to detect adulteration with BDG, the morphologically and genetically most similar adulterant. Recovery rates ranged from 81.79 to 102.38% in herbal mixed powders spiked with 1%, 5%, 50%, and 100% BDG. The method reliably detected QFT in processed tablets and freeze-dried decoctions, demonstrating high tolerance to deoxyribonucleic acid (DNA) degradation. This qPCR assay enables specific and quantitative detection of QFT in dried and processed samples using short amplicons (57 bp), thereby supporting quality control throughout the herbal production chain.

## 1. Introduction

In the Chinese Pharmacopoeia (Ch.P 2020), Qingfengteng (QFT) is defined as the dried vine stems of *Sinomenium acutum* (*S. acutum*) and *Sinomenium acutum* var. *cinereum* (*S. acutum* var. *cinereum*) [1]. Modern pharmacological studies have demonstrated that it possesses various activities, including anti-inflammatory, analgesic, anti-arrhythmic, and anti-tumor effects [2]. QFT decoction pieces and related preparations are widely used in the treatment of rheumatic diseases [3,4]. For example, BiKePian tablets, an exclusive formulation for rheumatoid arthritis, containing QFT as the primary component (accounting for approximately 30% by mass), combined with six other processed herbs [5].

QFT is distributed across most provinces in China, with major production bases located in Hunan, Hubei, Anhui, Henan, Shanxi, and Guizhou [6,7]. However, morphologically similar plants are frequently mislabeled as “QFT” in regional markets, including adulterants such as Beidougen (BDG, *Menispermum dauricum* DC.), Qingsheteng (QST, *Periploca calophylla* (Wight) Falc.), Jishiteng (JST, *Paederia foetida* L.), Kuanjinteng (KJT, *Tinospora sinensis* (Lour.) Merr.), and Huibeiqingfengteng (HBQFT, *Sabia discolor* Dunn) [8]. These adulterants are often derived from less frequently used medicinal herbs and have received limited scientific attention. To date, there have been no documented cases of clinical toxicity or adverse effects resulting from the misuse of these adulterants. However, these adulterants vary significantly in botanical origin, chemical composition, and therapeutic indications, which may pose risks to both efficacy and safety. Moreover, when these adulterants are processed into powders or preparations, they become visually indistinguishable, thereby introducing significant risks to medication. Consequently, QFT and its adulterants should not be mixed, and strict differentiation must be implemented in practical applications.

Current pharmacognostic studies have attempted to differentiate QFT from its adulterants using macroscopic and microscopic characteristics, while the observed differences are often insufficiently distinct [9]. Furthermore, traditional botanical identification methods are often highly subjective, experience-dependent, and significantly influenced by sample morphology, resulting in limited applicability due to interspecific variation and morphological similarities among related species. The reported deoxyribonucleic acid (DNA) barcoding, using the universal internal transcribed spacer 2 (ITS2) sequence for identifying QFT and its adulterants, could not handle heavily processed samples or quantitatively detect adulterants within mixed samples [8]. In contrast, after decades of refinement, mature real-time quantitative polymerase chain reaction (qPCR) technology is preferred for the molecular identification of traditional Chinese medicine (TCM) because it provides greater sensitivity for detecting highly processed products and low-concentration adulterants without the need for sequencing. Furthermore, it reduces detection time, enables high-throughput analysis, supports quantitative measurements, and ensures standardized experimental protocols with comparable results.

Thus, establishing an identification method with high specificity, accuracy, and robustness to physical variation is essential. qPCR enables real-time monitoring of DNA amplification via fluorescent signals proportional to amplicon yield, allowing for the quantification of target sequences. Owing to its high sensitivity, rapid cycling, real-time detection, high-throughput capacity, and quantitative capability, qPCR has been widely applied in food safety [10,11,12], microbiology [13,14,15], and the authentication of TCM [16,17], providing an effective tool for rapid species identification of easily confused herbs. As research on qPCR technology in TCM advances, expanding its applications and establishing a standardized evaluation system will enable more effective use of this method in both qualitative and quantitative studies.

Herein, we developed a TaqMan probe-based qPCR method for the specific detection of QFT in medicinal materials, decoction pieces, preparations, and common adulterants. This approach offers improved identification accuracy and provides novel strategies for enhancing quality control systems of QFT.

## 2. Materials and Methods

### 2.1. Instruments and Reagents

Real-time fluorescent quantitative PCR (qPCR-Analytik jena qTOWER3G, Jena, Germany; Roche LightCycler^®^480II, Basel, Switzerland; Apexbio Pangaea 6, Suzhou, Jiangsu, China); Analytical balance (Mettler AB135-S, Zurich, Switzerland); Ultrapure water systems (Millipore, Burlington, VT, USA); Ball mill (MM400, Retsch, Hamburg, Germany); Heating plate (IKA^®^ C-MAG HS7, Staufen, Germany); vacuum Freeze Dryer (BIOCOOL FD-1A-50, Beijing, China); NanoDrop One ultra-micro spectrophotometer (Thermo Fisher Scientific, Shanghai, China); Desktop high-speed freezing centrifuge (Eppendorf Centrifuge 5427 R, Leipzig, Germany); Qubit 4 Fluorometer (Thermo Fisher Scientific, Singapore); Fully automatic capillary electrophoresis nucleotide analyzer (Qsep100, BIOptic, New Taipei, Taiwan).

DNeasy mericon Food Kit (69514, Qiagen, Hilden, Germany); QIAquick^®^ Spin Columns (1112323, Qiagen, Hilden, Germany); Spin Columns (CapitalBio Technology, Beijing, China);Deoxynucleotide (dNTP) Solution Mix (10090191, BioLabs Inc., Ipswich, MA, USA); Q5^®^High-Fidelity DNA polymerase (M0491, Biolabs Inc., Ipswich, MA, USA); Probe qPCR Mix (2×) (RR392&RR391, TaKaRa, Shiga, Japan); Go Taq^®^ Probe qPCR Mix (A610A, PROMEGA, Madison, WI, USA); HQ Probe qPCR Mix (ZF601, Beijing Zhuangmeng International Biogene Technology Co., Ltd., Beijing, China); 6× MassRuler Dye (R0621, Fermentas, Vilnius, Lithuania); Polyvinylpyrrolidone (Jinkelong (Beijing) Biology Technology Co., Ltd., Beijing, China); Substitute of mercaptoethanol (Jinkelong (Beijing) Biology Technology Co., Ltd.); Sterilized distilled water.

### 2.2. Sample Collection and Identification

QFT medicinal materials were collected from wild sources, while their corresponding decoction pieces and adulterants were obtained from regional medicinal material markets. The pharmaceutical preparation (commercially named BiKePian in Chinese) was provided by the manufacturer. Processing methods and decoction times vary among manufacturers, with the maximum decoction time reaching 5 h. To evaluate the effect of decoction duration on DNA degradation in QFT, we simulated a traditional decoction process and laboratory-prepared freeze-dried powder samples with six different time periods (1 h, 2 h, 3 h, 4 h, 5 h, 6 h). All medicinal materials and decoction pieces were authenticated by pharmacognosy experts and through DNA barcoding. All sample information was listed in Table 1.

### 2.3. Preparation of the Freeze-Dried Powder of QFT Aqueous Decoction

Homemade aqueous decoction: Six equal-weight portions of QFT herb powder were weighed and decocted with 10-fold volumes of water for 1 h, 2 h, 3 h, 4 h, 5 h, and 6 h respectively (Sample NO. LAD01-06). The decoction was filtered, concentrated to a viscous consistency, and subsequently freeze-dried for 24 h with a vacuum freeze-dryer to obtain freeze-dried powder. Eventually, the obtained homemade aqueous decoction samples were subjected to subsequent DNA extraction and qPCR analysis.

### 2.4. DNA Extraction

The surfaces of dried medicinal materials and decoction pieces were wiped with 75% ethanol, and external impurities were removed using a double-edged razor blade. The middle sections were chopped into fragments and pulverized in a sterilized ball mill. Approximately 50 mg of the powdered material was weighed, and DNA was extracted using the DNeasy mericon Food Kit (Qiagen, Germany).

For pharmaceutical preparations and homemade aqueous decoctions, samples were washed sequentially with 75% ethanol and a pre-washing buffer (700 mM NaCl, 100 mM Tris-HCl [pH 8.0], 20 mM EDTA [pH 8.0], 0.2% PVP-40, and 0.4% β-mercaptoethanol), followed by centrifugation at 10,000 rcf for 5 min [18,19]. The supernatant was discarded, and the washing procedure was repeated until the supernatant appeared clear. DNA was then extracted from the pellet using the DNeasy mericon Food Kit.

DNA quality was assessed using a NanoDrop One ultra-micro spectrophotometer (Thermo Fisher Scientific, USA) and a Qubit 4 Fluorometer (Thermo Fisher Scientific, USA) with the dsDNA HS Assay Kit. The A260/A280 ratio was used to assess DNA purity and concentration. The absorbance ratios of all samples ranged from 1.6 to 2.0, with quality range from 360 to 2310 ng. All extracted DNA samples were stored at −20 °C.

### 2.5. ITS2 Sequence Analysis and Probe/Primer Design

The PCR amplification was performed in a 30 μL reaction mixture containing 0.2 μL forward primer (20 μmol/μL), 0.2 μL reverse primer (20 μmoL/μL), 0.2 μL Q5 High-Fidelity DNA polymerase, 0.6 μL dNTPs, 6 μL 5×reaction buffer, 1 μL DNA template and 21.8 μL sterile water. PCR Conditions: 98 °C for 30 s (initial denaturation), 40 cycles of 98 °C for 10 s (denaturation), 50 °C for 30 s (annealing) and 72 °C for 30 s (extension).

The ITS2 primers are obtained from the Ch.P (2020 edition, Volume IV, 9107). Forward (ITS2F) 5′-ATGCGATACTTGGTGTGAAT-3′,

Reverse (ITS2R) 5′-GACGCTTCTCCAGACTACAAT-3′. Amplified products were sequenced by AZENTA Life Sciences Biotechnology company. The obtained ITS2 sequences were trimmed and assembled using SeqMan software (v7.0.0), with primer regions removed. Reference ITS2 sequences for QFT and its adulterants were downloaded from the National Center for Biotechnology Information (NCBI) database. Standard sequences were aligned with detection sequence using MEGA software (v12.0.1.1). Based on nucleotide divergence, the closest relative adulterant (BDG), which exhibits the highest morphological similarity to QFT, was selected for comparative analysis. Species-specific probes and primers for QFT were designed using BioEdit software (v7.7.1.0). According to the differential nucleotide positions, probes were designed to be 20–30 bp in length with 40–70% GC content, and to yield amplicons less than 100 bp in length. The QFT-specific probe was labeled with 6-carboxyfuorescein (FAM) reporter dye at the 5′ end and black hole quencher (BHQ) at the 3′ end (Table 2).

### 2.6. Optimization of qPCR Amplification System

The initial amplification system consisted of 10 μL 2× Mix, 0.3 μL forward and reverse primer (0.3 μmol/μL), 0.3 μL probe (0.15 μmol/μL), 1.1 μL DNA template and 8 μL sterile water (total volume 20 μL). The initial PCR protocol was as follows: initial denaturation at 95 °C for 30 s, followed by 45 cycles of denaturation at 95 °C for 5 s, and annealing/extension at 60 °C for 15 s. Fluorescent signals were acquired at the 60 °C step of each cycle.

For optimization, we evaluated gradient annealing temperatures (59 °C to 63 °C, in 1 °C increments) and varying volumes of probe (0.3 μL, 0.6 μL) and primers (0.3 μL, 0.6 μL). Optimal parameters were selected based on amplification efficiency and specificity.

### 2.7. Specificity Test

DNA samples were amplified using the optimized qPCR system and the amplification products were analyzed using an automated capillary electrophoresis system (Qseq software, v3.4.3.0.6593) for molecular weight determination. The specificity of the probe and primers was validated by testing reference samples of QFT (QFT01) and adulterants (BDG01, JST01, KJT01, QST01, HBQST01) using the established extraction and amplification protocols.

### 2.8. Amplification Efficiency (E), Limit of Detection (LOD) and Limit of Quantification (LOQ)

A 10-fold serial dilution of DNA (QFT01) was prepared in deionized distilled water, yielding six concentrations (1.33 ng/μL to 0.0000133 ng/μL) for efficiency analysis.

The LOD was defined as the lowest DNA concentration yielding reproducible positive results. The LOQ was determined as the lowest concentration with relative standard deviation (RSD) ≤ 25% in quantitative measurements [19,20].

### 2.9. Repeatability and Robustness Test

Three replicate DNA samples were extracted from the standard medicinal material sample (QFT01). Under identical qPCR conditions, each DNA sample was assayed in triplicate to determine the cycle threshold (Ct) values, and the mean Ct value for each group was calculated. To assess robustness, different brands of premix solutions and fluorescent quantitative PCR instruments from various manufacturers were evaluated.

### 2.10. Applicability Test

The established qPCR method was used to detect QFT and its adulterants in samples from different habitats and batches. Ct values were recorded, and the method’s applicability was evaluated using coefficient of variation (CV) values, calculated as: CV (%) = (Standard deviation/Mean) × 100% (CV ≤ 25%).

### 2.11. Accuracy Test

Among the adulterants, BDG was selected as the research subject due to its high morphological similarity, close phylogenetic relationship to QFT, and prevalence in the medicinal material market.

Approximately 50 mg of mixed powders were prepared using standard medicinal materials (BDG01 and QFT01) at adulteration ratios of 0% (0/100), 1% (1/99), 5% (5/95) and 50% (50/50). All samples were processed for DNA extraction and qPCR amplification to obtain corresponding Ct values. The Ct values of QFT detected at different adulteration ratios was set as test sample (T) and pure sample (0% adulteration) was set as standard sample (S). The proportion of target DNA derived from QFT was calculated using the formula:

ΔCt = Ct (T) − Ct (S) (1)

Measured value = 2^−ΔCt^ × 100% (2)

Recovery rate (%) = (Measured value/Theoretical value) × 100%(3)

### 2.12. Statistical Analysis

Statistical analyses were performed using GraphPad Prism 9.5.0 (GraphPad Software, San Diego, CA, USA). This software generated linear fittings for standard curves and calculated coefficients of determination (R^2^). Data were imported into Microsoft Excel to compute mean values, standard deviations (SDs), relative standard deviations (RSDs), and coefficients of variation (CVs; calculated as SD/Mean).

## 3. Results

### 3.1. Specificity of Probes and Primers

**Figure 1:**
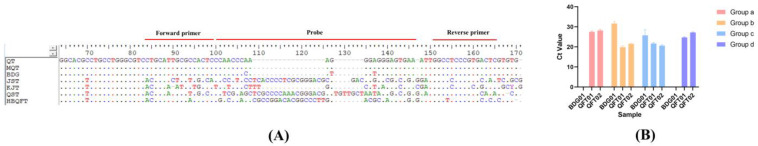
Probe and primer design and screen. (**A**) Location of probes and primers on ITS2 sequence designed for detecting QFT (QT: Qingfengteng (*Sinomenium acutum*); MQT: Qingfengteng (*Sinomenium acutum* var. *cinereum*); BDG: Beidougen; JST: Jishiteng; KJT: Kuanjinteng; QST: Qingsheteng; HBQFT: Huibeiqingfengteng). (**B**) Comparison of the effectiveness of four groups of probes and primers QFT detection (Group a: QFT-1_P; Group b: QFT-2_P; Group c: QFT-3_P; Group d: QFT-4_P; n = 3).

No nucleotide differences in the ITS2 sequences were observed between *S. acutum* and its variety *S. acutum* var. *cinereum*. Therefore, *S. acutum* was selected as the representative of QFT. In light of the differential nucleotide between QFT and BDG, four groups of specific probes and primers were designed (Figure 1A).

In group b and c, while QFT produced a detectable Ct value (CT < 35), BDG also yielded a Ct signal (false positive), indicating insufficient specificity and cross-reactivity. Nevertheless, compared with group a, group d exhibited superior specificity, with a low Ct value for QFT and no detectable signal for BDG (Figure 1B). Thus, group d was selected for subsequent qualitative and quantitative analyses of QFT and BDG.

### 3.2. Optimization of Annealing Temperature, Probe and Primer Concentration

**Figure 2:**
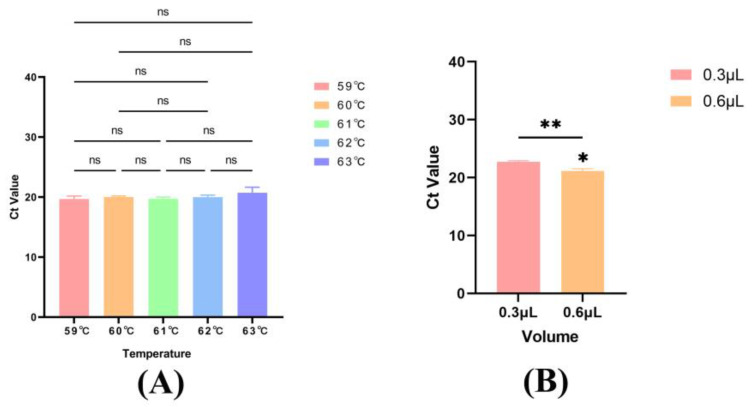
Amplification conditions applied for qPCR. (**A**) Annealing temperature (n = 3). (**B**) Volume of probes and primers (n = 3). ns represents not significant, * *p* < 0.05, ** *p* < 0.01.

Based on the established amplification protocol, six annealing temperatures (59 °C to 63 °C, in 1 °C increments) were evaluated. The results showed no statistically significant difference in Ct values across the temperature range (Figure 2A). Consequently, 60 °C, a commonly used annealing temperature, was selected for subsequent experiments.

In accordance with the original probe concentration of 0.15 μmol/μL, it increased to 0.30 μmol/μL for investigation. Similarly, the original primer concentration of 0.30 μmol/μL was increased to 0.60 μmol/μL for examination. When converted to the amplification system, both probe and primer volumes were adjusted to 0.6 μL. Comparative analysis demonstrated that employing 0.6 μL of both probe and primer resulted in a statistically significant difference (Figure 2B).

### 3.3. Specificity Evaluation

**Figure 3:**
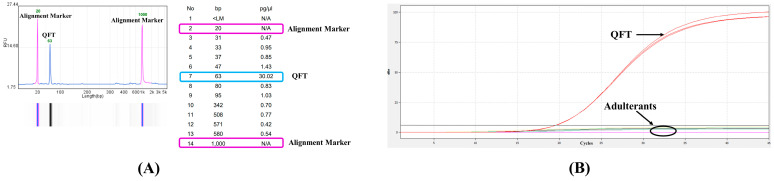
Specificity evaluation. (**A**) Molecular weight of qPCR amplification products by Qsep analyzer. (**B**) Amplification curve of target species and non-target species (target species: QFT, positive; non-target species: Adulterants, negative; n = 3).

Electropherogram analysis revealed a distinct single electrophoretic band with a corresponding singular peak, indicating a molecular size of 63 bp. The amplified nucleotide fragments exhibited high purity with no non-specific amplification or contaminant peaks, confirming the specificity of the PCR reaction (Figure 3A).

The amplification curve demonstrated that the qPCR system specifically amplified only the QFT target (positive result), while adulterants tested negative and remained undetected. These results validate the high specificity of the developed method, fulfilling the requirements for subsequent experiments (Figure 3B).

### 3.4. Amplification Efficiency (E), LOD and LOQ

**Figure 4:**
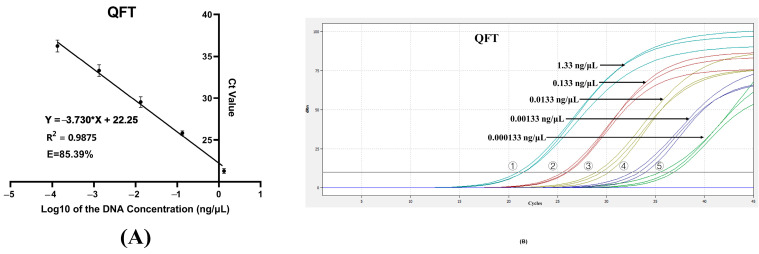
The evaluation of amplification efficiency and limit of detection. (**A**) Standard curve (R^2^ = 0.9875, slope = −3.730, E = 85.39%). Error bars represent standard deviation (n = 3). (**B**) Limit of detection (Line ①–⑤: DNA concentrations were 1.33 ng/μL, 0.133 ng/μL, 0.0133 ng/μL, 0.00133 ng/μL, 0.000133 ng/μL, respectively; n = 3).

A standard curve was generated by plotting Ct values (*y*-axis) against the logarithmic DNA concentrations (*x*-axis) using GraphPad Prism v9.5.0. The E was calculated from the slope of the curve. For the DNA concentration range 1.33 ng/μL to 0.000133 ng/μL, the regression equation was Y = −3.730x + 22.25 (R^2^ = 0.9875, E = 85.39%) (Figure 4A). The LOD was determined as 0.0001 ng/μL, the lowest concentration yielding a measurable Ct value (Figure 4B). The LOQ was identical to the LOD, as it was the minimum concentration with RSD ≤ 25% (Table 3). These results confirm the assay’s high sensitivity.

Currently, no unified statutory methodological guidelines exist for the application of qPCR in the field of TCM. This study adhered to performance criteria established by the European Network of Genetically Modified Organism Laboratories, along with relevant technical guidelines and literature, which recommend an amplification efficiency (E) of 90–110%, R^2^ ≥ 0.98, and a relative standard deviation (RSD) ≤ 25% at the limit of quantification (LOQ) [21,22,23]. TCM matrices are highly complex and variable, often containing inherent PCR inhibitors, such as polysaccharides and polyphenols, that suppress polymerase activity and reduce amplification efficiency. To better accommodate the practical challenges posed by such complex TCM matrices, the RSD threshold for the LOQ in this study was set at 25%. The observed amplification efficiency was 85.39%, slightly below the ideal range of 90–110%, while the R^2^ value of 0.9875 met the required criterion. Despite optimizations in DNA extraction methods and probe-primer design, amplification efficiency remained suboptimal. This limitation is likely attributed to persistent factors, including high levels of residual impurities in dried samples and the formation of primer dimers, both of which are inherently difficult to eliminate in complex TCM matrices. Nevertheless, acceptable recovery results confirmed that the detection accuracy remained satisfactory.

### 3.5. Repeatability and Robustness Analysis

Triplicate measurements of DNA extracts yielded Ct values of 18.53 ± 0.37, 18.39 ± 0.10, 18.61 ± 0.11 (Supplemental Table S1), demonstrating excellent reproducibility.

Comparative analysis revealed Ct ± SD value stability across commercial premix reagents (A610A: 23.72 ± 0.04, RR391: 23.09 ± 0.33, RR392: 21.37 ± 0.56) (Supplemental Table S2). Inter-instrument validation showed differential detection sensitivity (Roche: 18.97 ± 0.14, Analytik: 22.07 ± 0.36, Apexbio:16.46 ± 0.11) (Supplemental Table S3). All Ct values fell within an acceptable range, indicating high reliability and stability of the amplification method.

### 3.6. Applicability

47 batches of samples were detected in triplicate. Ct values for each sample were recorded. QFT exhibited positive amplification curves with Ct values (mean ± SD) ranging from 18.39 ± 0.10 to 20.88 ± 0.17 and CV values ranging from 0.10% to 2.37%, while adulterants showed negative results. The detection results were consistent with the specific identification results, indicating the qPCR method possessed robust applicability for the identification and detection of QFT and its adulterants (Table 4).

### 3.7. Accuracy Analysis

Mixed samples with BDG at different ratio (1%, 5% and 50%) were tested, and QFT (100%), BDG (0%) were served as positive and negative controls, respectively. The theoretical QFT content values were 99%, 95% and 50%. The measured values were 95.93%,97.27% and 40.90%, resulting in recovery rates of 96.90%,102.38% and 81.79%, respectively (Table 5). A standard curve was established by plotting ΔCt values against the theoretical value of QFT. The results demonstrated that the LOQ was 5% (RSD < 25%). The linear quantitative range was from 5% to 100%, with a regression equation of y = −2.6582x + 2.6142 (R^2^ = 0.996) (Supplemental Figure S1).

### 3.8. Analysis of BiKePian and Laboratory-Prepared Aqueous Decoctions Using qPCR

**Figure 5:**
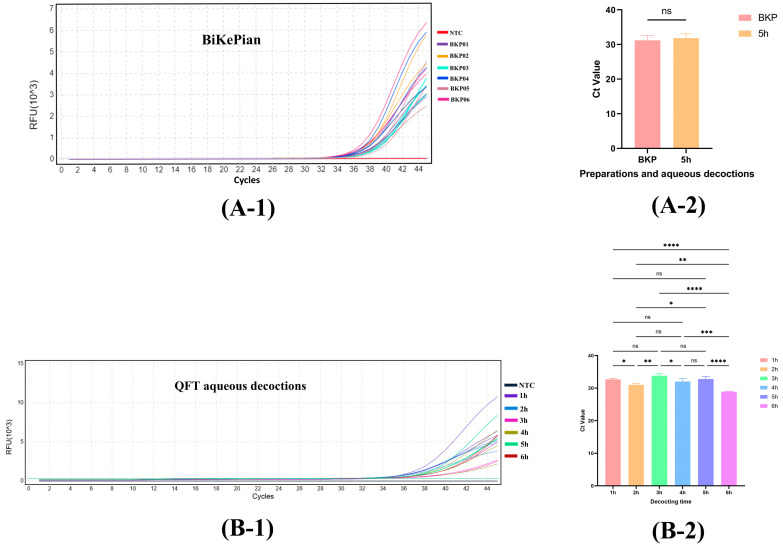
Bikepian and laboratory-prepared QFT aqueous decoctions. (**A-1**) Amplification curve of Bikepian preparations (n = 3). (**A-2**) Statistical comparison of BiKePian and laboratory-prepared QFT aqueous decoctions (n = 6). (**B-1**) Amplification curve of laboratory-prepared QFT aqueous decoction in six different decoction time (n = 3). (**B-2**) Statistical comparison of laboratory-prepared QFT aqueous decoctions with different decocting time (n = 3). ns represents not significant, * *p* < 0.05, ** *p* < 0.01, *** *p* < 0.001, **** *p* < 0.0001.

Six batches of BiKePian exhibited distinct amplification curves in real-time PCR, with Ct values (mean ± SD) ranging from 29.53 ± 0.33 to 33.09 ± 0.43, demonstrating the method’s effective detection of QFT in deeply processed tablets (Figure 5(A-1)). Statistical analysis was performed to compare BikePian and aqueous decoctions prepared with a 5-h decoction time (Figure 5(A-2)). The results demonstrated no significant difference in CT values, indicating that authentic QFT raw material was used and that the manufacturing process was consistent.

For laboratory-prepared aqueous decoctions of QFT raw material with varying decocting times (1 h to 6 h), all samples exhibited distinct amplification curves for the target DNA (Figure 5(B-1)). Regardless of decocting time, Ct values remained highly stable. Statistical analysis was conducted on aqueous decoctions prepared from six decoction time (Figure 5(B-2)). The results showed that the CT values of the aqueous decoctions were generally higher than those of the raw QFT medicinal materials or decoction pieces, indicating increased DNA degradation with prolonged decoction time. This observation was consistent with the overall upward trend in CT values.

Additionally, we performed agarose gel electrophoresis to characterize the DNA extracted from BiKePian and laboratory-prepared aqueous decoctions. Agarose gel electrophoresis experiment was performed on DNA extracted from the following ten samples: the DL 500 DNA Marker, blank control, QFT Medicinal material (non-decocted), laboratory-prepared freeze-dried powder samples with six different time periods (from left to right: 1 h, 2 h, 3 h, 4 h, 5 h, and 6 h), and the BiKePian (QFT preparation). The electrophoretogram revealed no discernible bands in the six aqueous decoctions samples and BiKePian, indicating substantial DNA degradation due to heat processing (Supplemental Figure S2). Nevertheless, the qPCR method developed in this study successfully detected DNA fragments from QFT, demonstrating its applicability for analyzing intensively processed products.

## 4. Discussion

QFT, a traditional Chinese medicine derived from vine stems, is susceptible to adulteration with morphologically similar species, posing potential safety risks in clinical applications. To date, reported molecular identification methods for QFT have relied solely on DNA barcoding, which is a time-consuming and qualitative technique incapable of detecting target species within mixed samples [8].

In this study, we analyzed ITS2 sequences from QFT and its adulterants (BDG, JST, KJT, QST, HBQFT). Basic local alignment search tool (BLAST) analysis of the amplicon was performed to evaluate the specificity of the assay against common adulterants. Based on regions of limited nucleotide divergence, species-specific TaqMan probes and primers were designed to target conserved interspecific sequences. The established qPCR method, grounded in ITS2 barcoding, incorporates optimized DNA extraction protocols for tablets samples, enhanced amplification systems, and rigorous validation procedures. This study focuses on the detection of QFT in mixed samples. Given that all adulterant species are derived from vine stems, they possess similar matrix properties and comparable DNA content. Therefore, the addition of other adulterants is not expected to significantly influence the quantitative results, demonstrating the high applicability of the developed method. Validation against potential unrecognized adulterants will be addressed in future studies. Compared to conventional DNA barcoding, the qPCR method developed herein offers several notable advantages: it utilizes shorter amplicons (<100 bp) and exhibits higher sensitivity, making it particularly suitable for detecting low-concentration adulterants in highly processed products. Moreover, unlike first-generation sequencing that requires pure samples, qPCR allows for the direct detection of mixtures. Additionally, it significantly reduces the detection time, enabling rapid on-site testing, and supports quantitative analysis.

This study establishes, for the first time, a TaqMan qPCR assay for highly specific identification of QFT-derived products. Validation confirmed exceptional specificity, sensitivity (LOD: 0.0001 ng/μL), applicability, repeatability (RSD ≤ 25%), and accuracy. The short-amplicon design (57 bp) enabled effective detection and quantification in dried and processed samples containing degraded DNA. Consistent with this method, most studies showed that shorter amplification fragments are more likely to be amplified successfully with better amplification efficiency and quantitative accuracy in highly processed materials [24,25,26,27,28]. Key advantages of short-amplicon assays include: (1) enhanced performance in highly degraded DNA samples; (2) shorter amplification time, enabling rapid on-site detection; and (3) reliable quantitative analysis. In contrast, DNA barcoding typically requires amplicons longer than 200 bp, and full-length ITS regions often exceed 500 bp. Such extended fragments are frequently amplified unsuccessfully in highly processed samples. Moreover, conventional sequencing-based methods are time-consuming and generally limited to qualitative applications rather than quantitative detection. Critically, the established qPCR method successfully detected QFT in both deeply processed tablets and laboratory-prepared decoctions. Tablets (e.g., BiKePian) The stable Ct values in decoctions boiled for 1–6 h demonstrated robust tolerance to DNA degradation in highly processed samples.

Although this qPCR method shows significant advantages for authenticating QFT, its quantitative application faces the following challenges. First, the quality of the extracted DNA is highly dependent on the extraction method, which may compromise quantitative accuracy. Second, detecting samples containing multiple adulterants requires further validation of the method’s applicability.

## 5. Conclusions

The qPCR method employed in this study demonstrated high sensitivity, detecting samples at concentrations as low as 0.0001 ng/μL and identifying adulteration at a 1% ratio. Due to the short amplicon length (57 bp), the method is also effective for analyzing highly processed products, including decoctions boiled for extended periods (up to 6 h) and commercial preparation. This work provides a reliable and efficient tool for authenticating QFT-derived raw materials and downstream products.

## Figures and Tables

**Table 1:** Information of the sample used in the study.

Dosage Form	Sample NO.	Latin Name	Place of Production/Collection
Medicinal materials	QFT01	*Sinomenium acutum* (Thunb.) Rehd. et Wils.	National Institutes for Food and Drug Control; Beijing, China
Decoction pieces	QFT02	*Sinomenium acutum* (Thunb.) Rehd. et Wils.	Anhui Jiren pharmaceutical Co., Ltd.; Bozhou, China
	QFT03	*Sinomenium acutum* (Thunb.) Rehd. et Wils.	Bozhou Chinese Medicine Exchange/Hubei; Bozhou, China
	QFT04	*Sinomenium acutum* (Thunb.) Rehd. et Wils.	Bozhou Chinese Medicine Exchange/Hubei; Bozhou, China
	QFT05	*Sinomenium acutum* (Thunb.) Rehd. et Wils.	Bozhou Chinese Medicine Exchange/Hubei; Bozhou, China
	QFT06	*Sinomenium acutum* (Thunb.) Rehd. et Wils.	Bozhou Chinese Medicine Exchange/Yuexi, Anqing, Anhui, China; Bozhou, China
	QFT07	*Sinomenium acutum* (Thunb.) Rehd. et Wils.	Bozhou Chinese Medicine Exchange/Enshi, Hubei, China; Bozhou, China
	QFT08	*Sinomenium acutum* (Thunb.) Rehd. et Wils.	Bozhou Chinese Medicine Exchange/Huaihua, Hunan, China; Bozhou, China
Medicinal materials	QFT09	*Sinomenium acutum* (Thunb.) Rehd. et Wils.	Fangxian, Shiyan, Hubei, China; Hubei, China
	QFT10	*Sinomenium acutum* (Thunb.) Rehd. et Wils.var. *cinereum* Rehd. et Wils.	Fangxian, Shiyan, Hubei, China; Hubei, China
Decoction pieces	QFT11	*Sinomenium acutum* (Thunb.) Rehd. et Wils.	Yuzhou Chinese Medicine Exchange/Hubei; Yuzhou, Henan, China
	QFT12	*Sinomenium acutum* (Thunb.) Rehd. et Wils.	Bozhou Chinese Medicine Exchange/Hubei; Bozhou, China
	QFT13	*Sinomenium acutum* (Thunb.) Rehd. et Wils.	Bozhou Chinese Medicine Exchange/Hubei; Bozhou, China
	QFT14	*Sinomenium acutum* (Thunb.) Rehd. et Wils.	Bozhou Chinese Medicine Exchange/Hubei; Bozhou, China
	QFT15	*Sinomenium acutum* (Thunb.) Rehd. et Wils.	Bozhou Chinese Medicine Exchange; Bozhou, China
	QFT16	*Sinomenium acutum* (Thunb.) Rehd. et Wils.	Bozhou Chinese Medicine Exchange; Bozhou, China
	QFT17	*Sinomenium acutum* (Thunb.) Rehd. et Wils.	Yulin Chinese Medicine Exchange/Hubei; Yulin, Guangxi, China
	QFT18	*Sinomenium acutum* (Thunb.) Rehd. et Wils.	Yulin Chinese Medicine Exchange/Hunan; Yulin, Guangxi, China
	QFT19	*Sinomenium acutum* (Thunb.) Rehd. et Wils.	Yulin Chinese Medicine Exchange/Hubei; Yulin, Guangxi, China
Medicinal materials	BDG01	*Menispermum dauricum* DC.	National Institutes for Food and Drug Control; Beijing, China
Decoction pieces	BDG02	*Menispermum dauricum* DC.	Bozhou Chinese Medicine Exchange; Bozhou, China
	BDG03	*Menispermum dauricum* DC.	Bozhou Chinese Medicine Exchange; Bozhou, China
	BDG04	*Menispermum dauricum* DC.	Bozhou Chinese Medicine Exchange; Bozhou, China
	BDG05	*Menispermum dauricum* DC.	Bozhou Chinese Medicine Exchange/Heilongjiang; Bozhou, China
	BDG06	*Menispermum dauricum* DC.	Bozhou Chinese Medicine Exchange; Bozhou, China
	BDG07	*Menispermum dauricum* DC.	Yulin Chinese Medicine Exchange/Hubei; Yulin, Guangxi, China
	BDG08	*Menispermum dauricum* DC.	Yulin Chinese Medicine Exchange; Yulin, Guangxi, China
	KJT01-05	*Tinospora sinensis* (Lour.) Merr.	Bozhou Chinese Medicine Exchange; Bozhou, China
	JST01-05	*Paederia foetida* L.	Bozhou Chinese Medicine Exchange; Bozhou, China
	HBQFT01-05	*Sabia discolor* Dunn	Yulin Chinese Medicine Exchange/Guilin; Yulin, Guangxi, China
	QST01-05	*Periploca calophylla* (Wight) Falc.	Hehuachi Chinese Medicine Exchange/Zhejiang; Chengdu, Sichuan, China
Preparations	BKP01-06	/	Hefei Jinyue Pharmaceutical Co., Ltd.; Hefei, China
Laboratory-prepared QFT aqueous decoctions	LAD01-06		Laboratory-prepared; Beijing, China

QFT: Qingfengteng. BDG: Beidougen. KJT: Kuanjinteng. JST: Jishiteng. HBQFT: Huibeiqingfengteng. QST: Qingsheteng. BKP: BiKePian. LAD: Laboratory-prepared QFT aqueous decoctions.

**Table 2:** The sequences of probes and primers designed for this study.

NO.	Sequence (5′→3′)	Primers and Probe	Length (bp)	Detection Target
a	FAM-TCCCAACCCAAAGGGAGGGAGTG-BHQ	QFT-1_P	55	ITS2 regions of QFT
	CCTGCATTGCGCCAC	QFT-1_F		
	GTCACGGGAGGCCAATT	QFT-1_R		
b	FAM-TTGCCATCGAGGGTAAATTGAACCCTTGT-BHQ	QFT-2_P	77	
	GAAATAGGATGACTTGATCGAGTAG	QFT-2_F		
	TGGGGTCGCATGGTATATG	QFT-2_R		
c	FAM-CCATCGAGGGTAAATTGAACCCTTGTAGCT-BHQ	QFT-3_P	73	
	TAGGATGACTTGATCGAGTAGTTG	QFT-3_F		
	TGGGGTCGCATGGTATATG	QFT-3_R		
d	FAM-CCAACCCAAAGGGAGGGAGTGAA-BHQ	QFT-4_P	57	
	CTGCATTGCGCCACTC	QFT-4_F		
	GAGTCACGGGAGGCC	QFT-4_R		

**Table 3:** LOD and LOQ validation of this method (n = 3).

DNA Concentration (ng/µL)	Ct (Mean ± SD)	RSD (%)
1.33	21.32 ± 0.28	1.31%
0.133	25.84 ± 0.28	1.08%
0.0133	29.54 ± 0.64	2.17%
0.00133	33.30 ± 0.69	2.07%
0.000133 (LOD, LOQ)	36.24 ± 0.71	1.96%
0.0000133	ND (Not detected)	

**Table 4:** Applicability of 47 bathes of QFT and adulterants from different sources (n = 3).

	QFT				Adulterants		
NO.	Ct (Mean ± SD)	CV (%)	NO.	Ct (Mean ± SD)	CV (%)	NO.	Ct
QFT01	18.39 ± 0.10	0.54	QFT11	18.45 ± 0.17	0.92	BDG01~08	-
QFT02	19.00 ± 0.45	2.37	QFT12	19.20 ± 0.23	1.20	JST01~05	-
QFT03	19.44 ± 0.33	1.70	QFT13	19.37 ± 0.36	1.86	KJT01~05	-
QFT04	19.97 ± 0.09	0.45	QFT14	19.34 ± 0.19	0.98	QST01~05	-
QFT05	19.56 ± 0.02	0.10	QFT15	20.71 ± 0.16	0.77	HBQFT01~05	-
QFT06	20.02 ± 0.34	1.70	QFT16	19.50 ± 0.06	0.31		
QFT07	19.64 ± 0.27	1.37	QFT17	19.05 ± 0.04	0.21		
QFT08	19.29 ± 0.08	0.41	QFT18	20.81 ± 0.12	0.58		
QFT09	20.88 ± 0.17	0.81	QFT19	19.14 ± 0.39	2.04		
QFT10	19.32 ± 0.10	0.52					

**Table 5:** Accuracy evaluation with standardized mixed samples (n = 3).

Theoretical Value of QFT (%)	Ct (Mean ± SD)	Measured Value (%)	Recovery (%)
50%	19.90 ± 0.25	40.90%	81.79%
95%	18.65 ± 0.15	97.27%	102.38%
99%	19.03 ± 0.41	95.93%	96.90%
100%	18.61 ± 0.11		

## Data Availability

The data supporting this study have been deposited in a public repository: Experimental raw data: Repository: Figshare DOI: 10.6084/m9.figshare.30127267.v1.

## Supplementary Materials

The following supporting information can be downloaded at: https://www.mdpi.com/article/10.3390/molecules30183763/s1, Table S1. Result of repeatability (n = 3, *p* > 0.05). Table S2. Investigation of different premixed solutions (n = 3, *** *p* < 0.001). Table S3. Investigation of different fluorescent quantitative PCR instruments (n = 3, **** *p* < 0.0001). Figure S1. Normalized calibration curves achieved by qPCR targeting QFT using the ΔCt method (ΔCt = Ct (T) − Ct(S)) and reference mixtures of adulterant BDG. Figure S2. Agarose gel electrophoresis image of DNA extracted from laboratory-prepared aqueous decoctions and BiKePian, 1: DL 500 DNA markers, 2: Blank control, 3: QFT Medicinal material (non-decocted), 4–9: Laboratory-prepared freeze-dried powder samples with six different time periods (From left to right: 1 h, 2 h, 3 h, 4 h, 5 h, 6 h), 10: BKP (QFT preparation).

## Abbreviations

Chinese pharmacopoeia, (Ch.P); *Sinomenium acutum*, (*S. acutum*); *Sinomenium acutum* var. *cinereum*, (*S. acutum* var. *cinereum*); the real-time fluorescent quantitative PCR, (qPCR); traditional Chinese medicine, (TCM); second internal transcribed spacer, (ITS2); National Center for Biotechnology Information, (NCBI); Polyvinylpyrrolidone, (PVP); Limit of detection (LOD); limit of quantification (LOQ).
