# Supplementary material for: Development of TaqMan Real-Time Fluorescent Quantitative PCR Method for Identification and Quantification of Sinomenium acutum-Originated Herbal Drugs
Source: Molecules. 2025 Sep 16;30(18):3763. doi: 10.3390/molecules30183763 (PMC12472803; doi:10.3390/molecules30183763)
Supplement: Supplementary file 1 [file molecules-30-03763-s001.zip › molecules-3765901-supplementary.pdf]

Table S1. Result of repeatability (n = 3, $p > 0.05$).

NO.	Ct			
	1	2	3	Mean ± SD
1	18.37	18.95	18.26	18.53±0.37
2	18.48	18.39	18.29	18.39±0.10
3	18.72	18.62	18.51	18.61±0.11

Table S2. Investigation of different premixed solutions (n = 3, ***$p < 0.001$).

Premixed solution	Ct			
	1	2	3	Mean ± SD
RR391	22.77	23.07	23.44	23.09±0.33
RR392	20.79	21.43	21.90	21.37±0.56
A610A	23.68	23.75	23.74	23.72±0.04

Table S3. Investigation of different fluorescent quantitative PCR instruments (n = 3, ****$p < 0.0001$).

Instrument brand	Ct			
	1	2	3	Mean ± SD
Roche LightCycler®480II	18.81	19.05	19.06	18.97±0.14
Analytik jena qTOWER3G	22.36	21.66	22.19	22.07±0.36
Apexbio Pangaea 6	16.46	16.32	16.60	16.46±0.11

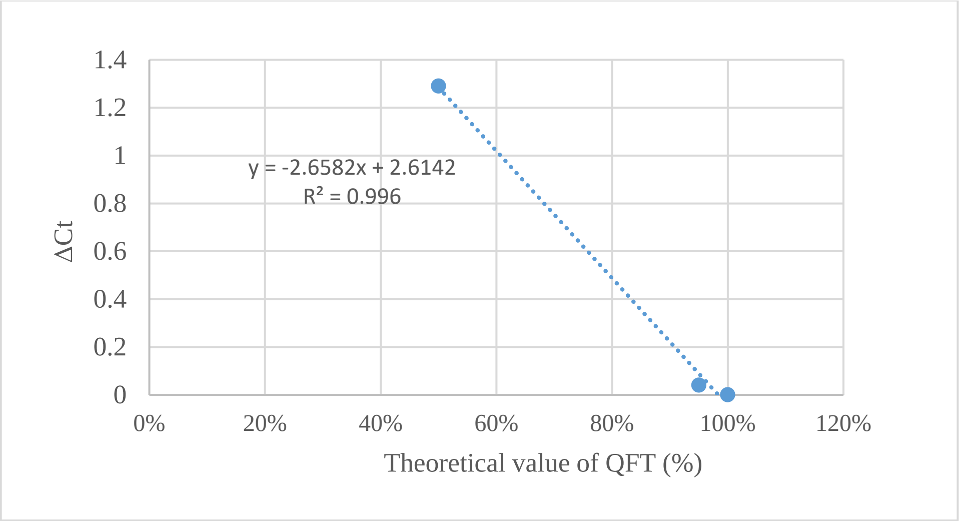

Figure S1. Normalized calibration curves achieved by qPCR targeting QFT using the ΔCt method (ΔCt=Ct (T)- Ct(S)) and reference mixtures of adulterant BDG.

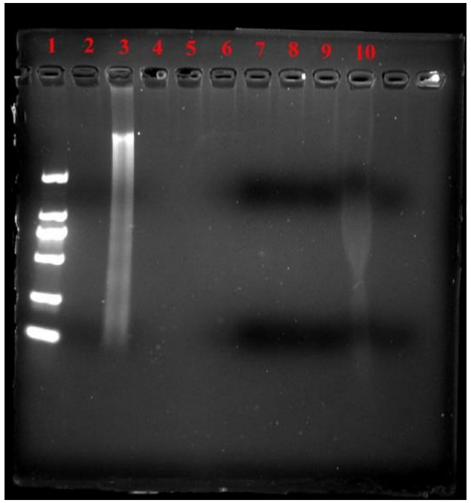

Figure S2. Agarose gel electrophoresis image of DNA extracted from laboratory-prepared aqueous decoctions and BiKePian, 1: DL 500 DNA markers, 2: Blank control, 3: QFT Medicinal material (non-decocted), 4-9: Laboratory-prepared freeze-dried powder samples with six different time periods (From left to right: 1 h, 2 h, 3 h, 4 h, 5 h, 6 h), 10: BKP (QFT preparation).
